# Protein adsorption onto nanoparticles induces conformational changes: Particle size dependency, kinetics, and mechanisms

**DOI:** 10.1002/elsc.201500059

**Published:** 2015-11-10

**Authors:** Peter Satzer, Frantisek Svec, Gerhard Sekot, Alois Jungbauer

**Affiliations:** ^1^ Department of BiotechnologyUniversity of Natural Resources and Life SciencesViennaAustria; ^2^ Lawrence Berkeley National LaboratoryThe Molecular FoundryBerkeleyCAUSA; ^3^ Austrian Centre of Industrial Biotechnology (ACIB)ViennaAustria

**Keywords:** Adsorption, Conformational change, Curvature, Nanoparticles, Particle size

## Abstract

The use of nanomaterials in bioapplications demands a detailed understanding of protein–nanoparticle interactions. Proteins can undergo conformational changes while adsorbing onto nanoparticles, but studies on the impact of particle size on conformational changes are scarce. We have shown that conformational changes happening upon adsorption of myoglobin and BSA are dependent on the size of the nanoparticle they are adsorbing to. Out of eight initially investigated model proteins, two (BSA and myoglobin) showed conformational changes, and in both cases this conformational change was dependent on the size of the nanoparticle. Nanoparticle sizes ranged from 30 to 1000 nm and, in contrast to previous studies, we attempted to use a continuous progression of sizes in the range found in live viruses, which is an interesting size of nanoparticles for the potential use as drug delivery vehicles. Conformational changes were only visible for particles of 200 nm and bigger. Using an optimized circular dichroism protocol allowed us to follow this conformational change with regard to the nanoparticle size and, thanks to the excellent temporal resolution also in time. We uncovered significant differences between the unfolding kinetics of myoglobin and BSA. In this study, we also evaluated the plausibility of commonly used explanations for the phenomenon of nanoparticle size‐dependent conformational change. Currently proposed mechanisms are mostly based on studies done with relatively small particles, and fall short in explaining the behavior seen in our studies.

**Abbreviations**
CD: circular dichroism
DLS: dynamic light scattering
TEM: transmission electron microscopy

## 1 Introduction

A fundamental understanding of surface curvature effects on the adsorption of biomolecules is important for the development of new surfaces and nanomaterials. These novel materials may be employed in diverse fields, from transport, where they can be used for surface modifications of ship hulls to prevent biofouling, to biomedical engineering, where they allow drug delivery to tumor cells or are used as adjuvants in vaccines 1, 2, 3. Spherical nanoparticles also made significant contributions to new healthcare applications such as vaccination, 4 cancer treatment, and imaging 5, 6. Nanoparticles exhibit unique behavior in the human body, but there is only a limited knowledge of how nanomaterials interact with cells and proteins. Depending on the nanoparticle size and surface chemistry, proteins can form a corona at the external surface of nanoparticles, when administered to the human body 7, 8, 9, 10. The specific protein corona of differently functionalized or differently sized nanoparticles influence all aspects of nanoparticle–organism interaction ranging from cytotoxicity to uptake kinetics 11, 12, 13. Nanoparticles that enter a physiological environment are covered by protein almost instantaneously. Not only the actual surface of the nanoparticle dictates its behavior in vivo, but also the proteins attached to the nanoparticle. Adsorbing proteins in their natural conformation, or forcing them to change their conformation upon adsorption could make the difference between an effective drug carrier and a toxic nanoparticle. Thus, understanding the specific protein–nanoparticle interactions and how or why proteins might change their conformation upon adsorption is key to comprehend how the nanoparticle surfaces behave in vivo. Typically, proteins can change their conformation upon binding to surfaces and this behavior is affected by the type of surface and surface geometry, in some cases these interaction might be specific for the given protein–nanoparticle interaction 14, 15, 16, 17, 18, 19, 20. In recent years, an increasing number of studies described size‐dependent protein denaturation by nanomaterials (see reviews 21, 22, 23) but the results were not conclusive. A comprehensive list of materials, proteins, and methods used in relevant sources can be found in the Supporting information, Table S1. One study reports increasing conformational change on smaller particles 24. However, most studies conclude that conformational changes are more substantial with larger particles 25, 26, 27, 28. One report finds stabilization of the secondary structure 29. None of the reports give a comprehensive dataset that would allow a detailed investigation of the underlying mechanisms, as they only report the behavior on up to three nanoparticle sizes. A dataset so small is not able to provide sufficient data to follow the conformational changes in terms of protein size accurately. Therefore, such a dataset is not sufficient to test the proposed mechanisms for plausibility. Many of these reports propose as mechanism that the surface curvature itself may force bending of the protein. Alternative explanations assume that nanoparticles with less surface curvature may provide more area for interaction with the protein. Another explanation relies on the difference in the geometry‐dependent double‐layer potential that forms at the particle surface 26. All of these studies lack the data necessary to evaluate the proposed mechanisms, because of the limited number of nanoparticle sizes used in the investigations. Compiling these results into one big picture for the evaluation of these hypotheses is impossible, since the studies present a limited number of particle sizes, different proteins, and different nanoparticle materials.

In this work, we provide a dataset of interactions between model proteins with a wide range of nanoparticle sizes. This conclusive dataset can in turn be used to evaluate explanations commonly offered for this phenomenon.

## 2 Materials and methods

All chemicals and materials were purchased from Sigma–Aldrich (St. Louis, USA) unless indicated otherwise.

### 2.1 Nanoparticles

Silica nanoparticles with amidine surface modification with a size of 30, 70, 100, 150, 200, 300, 500, and 1000 nm were purchased from Kisker (Steinfurt, Germany). Their size and monodispersity was confirmed by transmission electron microscopy (TEM) and dynamic light scattering (DLS). Their nonporosity was confirmed by nitrogen adsorption and the surface charges were characterized by their zeta potential.

### 2.2 Determination of adsorption isotherm

Protein solutions of concentrations up to 4 mg/mL in 20 mM phosphate buffer, pH 7 with and without addition of 1 M NaCl were mixed with a nanoparticle dispersion to reach a concentration of 2.5 mg/mL for nanoparticles smaller than 100 nm, and 5 mg/mL for nanoparticles 150 nm and larger. The mixtures were incubated for 12 h to reach equilibrium. Particles were removed by filtration through 100 kDa membranes (Merk Millipore, Billerice, USA). The concentration of residual protein in solution was determined using UV absorption at a wavelength of 280 nm. All measurements were done in triplicate.

### 2.3 Desorption of protein

Desorption of proteins from nanoparticles was tested by using the particles from adsorption isotherm experiments and suspending them to 20 mM phosphate buffer pH 7 either with the addition of 1 M NaCl or without the addition of salt and incubated them for 12 h. Desorbed proteins were measured after 12 h of incubation using UV adsorption at a wavelength of 280 nm. Samples transferred into 20 mM phosphate buffer pH 7 without NaCl were used as a control.

### 2.4 Determination of size, zeta potential, and polydispersity of nanoparticles

DLS of nanoparticle dispersions in a 20 mM phosphate buffer pH 7 was carried out using a Zetasizer Nano S (Malvern Instruments, Malvern, UK). Particle and solution parameters (refractive index and viscosity) were obtained from the library that came with the instrument. All measurements were performed in triplicate.

### 2.5 Circular dichroism

All measurement were carried out using a JASCO J‐1100 instrument (JASCO, Easton, USA) with a quartz‐cuvette and a 20 mM phosphate buffer pH 7, with recording wavelength from 190 to 240 nm. A concentration of 1 mg/mL was used for determination of the protein structure of unbound protein. Bound protein structures were determined after coating the particles with protein at 1.5 mg/mL particle solution by using 4 mg/mL protein and subsequent removal of the unbound protein. The protein‐coated nanoparticles were washed three times with fresh buffer solution to remove any residual unbound protein. The washed protein‐coated nanoparticles were measured from 190 to 240 nm in a 1 mm path length cuvette. The concentration of adsorbed proteins was taken from the adsorption isotherms experiments. The spectra were blank subtracted with protein‐free nanoparticle solution spectra. Spectra were measured ten times and condensed into a single spectrum before structure determination to reduce noise.

Experiments to show differences in the structure of adsorbed protein for high and low surface coverage of the particle were done with a particle concentration of 1.5 mg/mL and either 4 mg/mL of protein or 0.01 mg/mL of protein washed and measured as described in this section. Time‐dependent circular dichroism (CD) measurements were carried out with a particle concentration of 1.5 mg/mL and protein concentration of 0.01 mg/mL. This ensures that almost all protein present in the solution is bound to the nanoparticle and a washing step can be omitted. Because of decreased signal‐to‐noise ratio, these experiments were not performed for 500 or 1000 nm particles.

The structure of proteins was either calculated according to Raussens et al. 30 without using the protein concentration, or was calculated using the K2D3 software 31, using concentration values obtained from the adsorption isotherm experiments.

## 3 Results and discussion

### 3.1 Nanoparticle characterization

**Figure 1:**
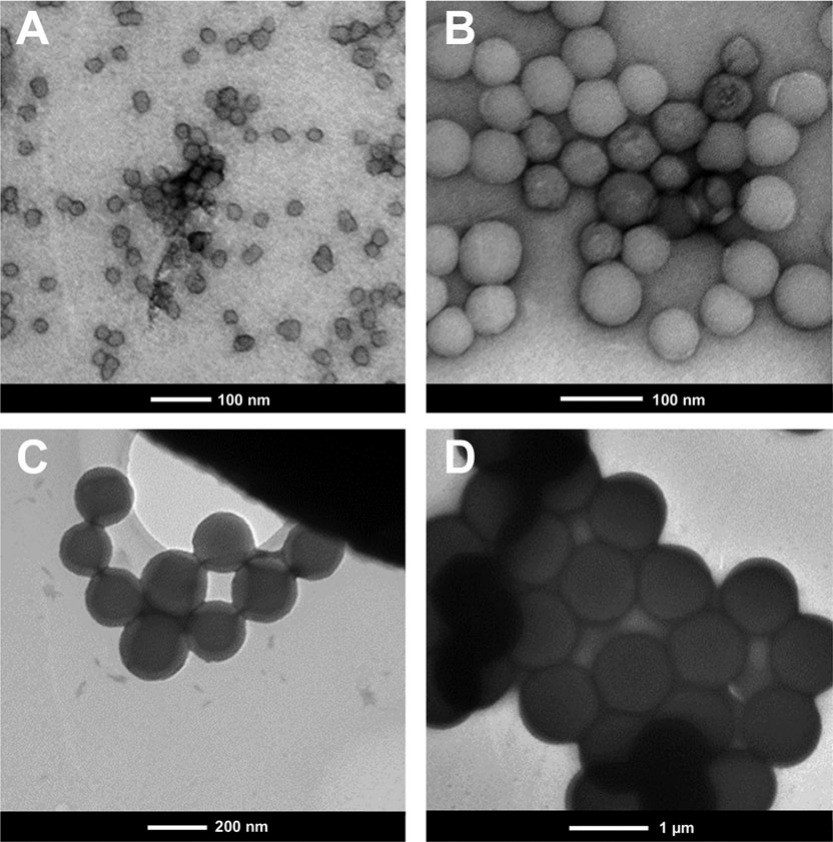
TEM images of spherical silica nanoparticles show monodispersity and spherical shape showing (A) 30 nm particles, (B) 70 nm particles, (C) 200 nm particles, and (D) 1000 nm particles.

The nanoparticles used for this study were characterized thoroughly to be sure that the material provided by the manufacturer is suitable for this study. Factors of interest were particle shape, surface roughness, porosity, and the zeta potential. We investigated amidine‐modified particles of a nominal size of 30, 70, 100, 150, 200, 300, 500, and 1000 nm by TEM and DLS to determine size and polydispersity (Fig. 1 shows TEM pictures; TEM and DLS results are summarized in Table 1). Sizes determined by TEM and DLS were in good agreement to the nominal sizes. The monodispersity and spherical shape could also be confirmed.

**Table 1:** List of nanoparticles used in this study and their characteristics

Particle diameter (nm)					Surface area (m²/g)	
Nominal	TEM	DLS	PDI^a^^)^	ζ (mV)^b^^)^	Calculated	Measured
30	23.1	26.5	0.147	−46.9	130.6	ND^c^^)^
70	62.9	65.1	0.025	−62.7	48.3	51.2
100	ND	110.7	0.029	−64.6	27.5	ND
150	ND	155.2	0.017	−66.2	20.1	ND
200	192.4	213.6	0.009	−70.4	18.3	20.2
300	ND	302.9	0.021	−72.1	10.0	ND
500	ND	460.8	0.041	−71.1	7.2	ND
1000	1022.2	1074.1	0.049	−70.1	3.1	3.5

a Polydispersity index obtained from DLS measurement. The lower the number, the narrower the dispersity.

b Zeta potential.

c Not determined.

The surface properties were assessed by measuring the zeta potential, characterizing the charges present on the surface. The results of these measurements are presented in Table 1 and all zeta potentials are highly negative and deviate only slightly from each other with the exception of very small particles. The zeta potential for particles above 150 nm can be considered as not changing at all; For smaller particles we have to keep the differences in mind, especially for the very small particles of 30 nm.

The last property to investigate was the porosity of the particles since porosity would make our results based on the assumption of solid particles useless. We determined surface areas of 70, 200, and 1000 nm nanoparticles through nitrogen adsorption experiments and the results are in good agreement with the theoretical surface calculated for solid spheres of the same size, indicating no porosity as well as no significant surface roughness (Table 1).

### 3.2 Protein characterization

We used nine proteins in the binding studies that differed in their properties as shown in Supporting information, Table S2. First, we investigated the binding of all proteins to 70 nm nanoparticles at pH 7 in 20 mmol/L phosphate buffer (Supporting information, Table S2). No binding was observed for glucose oxidase and also for chymotrypsin. We continued the study using the remaining seven model proteins and determined their structure in solution (Supporting information, Table S3). The structure determination was done by a simple method presented by Raussens et al. 30. The method is susceptible to systematic errors in the quantification of secondary structure elements, but remains sensitive to changes.

### 3.3 Conformational changes of proteins

We determined the structural parameters of each of the proteins before and during contact with nanoparticles. Several previous studies measured the changes for protein after contact with the nanoparticle, but not in its adsorbed state 22, 32, 33. Lundqvist et al. 32, 33, 34, 35 investigated proteins by NMR bound to very small nanoparticles with two different proteins and we attempted to build onto and expand this work, especially to nanoparticle sizes similar to that of viruses. NMR is not applicable for large particles, and as such an alternative method was sought. CD spectroscopy was found to be applicable for the structural prediction required in this study. To counteract the additional noise introduced by nanoparticles obstructing the light path, we chose to measure the CD spectra at very high concentrations of adsorbed protein. We removed unbound proteins by washing the particles, and enhanced the signal by condensing ten individual spectra into one measurement for structure determination (a representative CD spectra used for structure determination can be seen in Supporting information, Fig. S1). The protein concentration on the particles was not determined in these first experiments and structures were estimated by the use of a concentration‐independent method 30. Since this method has its limitations, it was only used for a fast screening of our model proteins.

We observed that the seven model proteins can be grouped in three categories: (i) proteins that do not reveal any conformational change, (ii) proteins for which the conformational change was unclear due to signal/noise, and (iii) proteins that exhibit clear conformational change.

The first group included lysozyme, β casein, and ribonuclease A. These proteins showed no conformational change after adsorption on nanoparticles of any size (Supporting information, Fig. S2). This result was expected as two of the proteins are very stable (ribonuclease A and lysozyme) and one of them lacks a very pronounced secondary structure (β casein).

The second category of proteins included cytochrome C and ovalbumin. The data we collected are not sufficient to judge the unfolding of the protein upon adsorption (Supporting information, Fig. S3). Our data may indicate a change in the alpha‐helical content in the protein structure depending on the nanoparticle size. However, the experimental error of the method used is too big to make any reliable conclusions.

**Figure 2:**
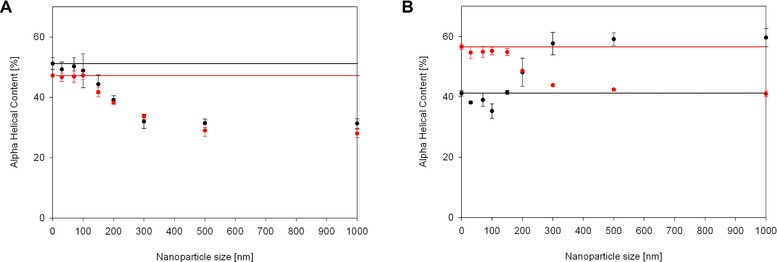
Alpha‐helical content of (A) myoglobin and (B) BSA adsorbed on nanoparticles varying in sizes. The red curve shows the concentration‐dependent structure prediction by Louis‐Jeune et al. 31, the black curve shows the concentration‐independent structure prediction by Raussens et al. 30. Horizontal lines represent the structure of the protein in solution. A clear difference between the two methods in the structure prediction for BSA is observable, whereas both methods are in excellent agreement for myoglobin. Ten individual spectra were condensed into one before structure determination. Three of such sets were used to calculate SDs shown as error bars in the figure.

The proteins that showed clear conformational changes were myoglobin and BSA, both alpha‐helical rich proteins. We determined adsorption isotherms for all nanoparticle sizes for these two proteins to estimate the amount of bound protein (Supporting information, Fig. S4). Concentrations taken from these curves were used to determine the secondary structure composition by the K2D3 software 36. Both proteins show a decrease in alpha‐helical structure once adsorbed to relatively large particles above 150 nm in size (Fig. 2). Interestingly the structure prediction given by the concentration‐independent method predicted an increase in alpha‐helical structure of BSA on larger particles, rather than a decrease. This clearly demonstrates the limitation of concentration‐independent structure determination, as it is sensitive to changes, but does not give exact values. In fact, in this case it inversed the observed trend.

To be sure we are dealing with significant differences between the small and large particles, we performed a *t*‐test including all measurements from 0 to 100 nm into one group and all measurements from 300 to 1000 nm into another group and tested if the two population are significantly different. For myoglobin and for BSA the resulting *p*‐value is below 0.0001, meaning the difference is of very high statistical significance.

A very interesting detail is the sigmoidal shape of the curve in both cases, which gives the opportunity to investigate proposed mechanisms for this denaturation. Something has to change significantly between the particle size of 100 and 300 nm. Looking at this window, we can exclude the different zeta potentials found in our particle analysis (Table 1) as driving factor for the conformational change. If the zeta potential would be the driving force, we would expect a difference between 30 nm particles and the rest as well as no change above 150 nm particle size.

**Figure 3:**
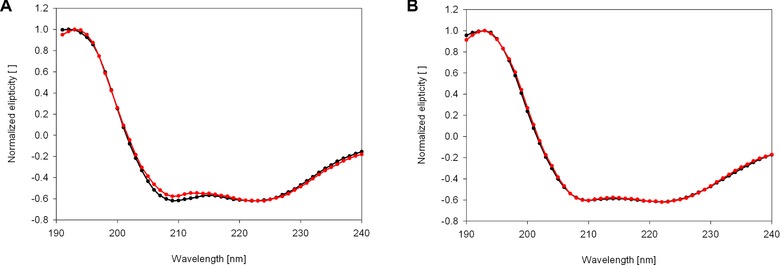
Normalized spectra of (A) myoglobin and (B) BSA adsorbed at low concentration (black line) and high concentration (red line). A small concentration‐dependent conformation change is visible in myoglobin, but not in BSA.

To quantify the effect of concentration‐dependent changes of conformation we calculated the protein load per surface area for the particles in this experiment (Table 2). We can clearly see that the protein load on this particles rises between 30 and 70 nm particle size, stays relatively stable in the region of 70–200 nm and then again rises as the particles grow bigger. Such a trend of increased BSA adsorption onto less curved surfaces was also recently shown by experiment and simulation in the case of carbon nanotubes 37. Rough calculations using as protein size 2 and 4 nm for myoglobin and BSA, respectively, show that on all particle sizes the adsorption is a thick multilayer. The calculated coverage ranged from 700 to 7000% of the available surface area. We do not know why the multilayer increases with the nanoparticle size, but the unfolding of the protein may be a driving force for building up huge multilayers. This might expose hydrophobic regions at the surface where other protein molecules might attach. To test for concentration‐dependent conformational changes we measured the structure of adsorbed proteins at very low and very high surface concentration on 300 nm nanoparticles. The normalized spectra (Fig. 3) show no difference in the spectra for high and low concentration for BSA, but show a small change in the spectra for myoglobin corresponding to a change in alpha‐helical content of 3%.

**Table 2:** Adsorbed protein amount for myoglobin and BSA at a protein concentration of 4 mg/mL

Particle size (nm)	Adsorption capacity (g/m²)	
	Myoglobin	BSA
30	0.60	0.76
70	0.98	1.13
100	0.80	1.70
150	0.98	1.74
200	1.08	1.80
300	1.83	3.51
500	2.51	3.88
1000	5.48	6.02

### 3.4 Kinetics of adsorption and conformational change

**Figure 4:**
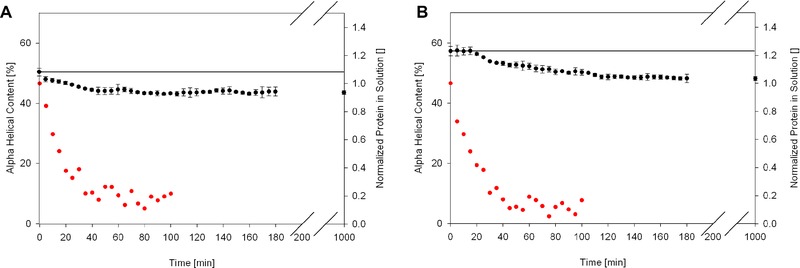
Kinetics of adsorption kinetic (red) and conformational changes after adsorption (black). (A) Myoglobin on 200 nm silica nanoparticles and (B) BSA on 300 nm silica nanoparticles. Kinetics of adsorption and conformational change happen simultaneously for myoglobin, but not for BSA. Ten individual spectra were collected and used to calculate SDs shown as error bars in the figure.

We used CD spectroscopy to follow the conformational change of myoglobin and BSA because we wanted to know if the unfolding or the adsorption is the rate limiting step. To get reasonable results we had to modify the previous experimental setup. The washing and dissolution steps used before require an extra hour of sample handling, which of course makes it impossible to measure kinetics. To overcome this limitation we used a low protein concentration setup that does not require a washing step. To further increase the signal/noise ratio we only investigated the smallest particles that showed significant structural changes in previous experiments: 200 nm particles for myoglobin and 300 nm particles for BSA. Adsorption kinetic experiments showed the adsorption of protein in both cases to be a relatively fast process. The adsorption was finished in 30 min (Fig. 4).

Figure 4 shows the time trace of the alpha‐helical structure of myoglobin (panel A) and BSA (panel B) determined by the K2D3 software. Myoglobin seems to undergo a fast conformational change, finished in about the same duration as the adsorption, whereas BSA takes longer to change its conformation. Looking at these results we can conclude that myoglobin contradicts the usual assumption that conformational changes are a slow process compared to adsorption. Myoglobin adsorbs and almost immediately changes its conformation. BSA follows the classical principle as the rate limiting step is the conformational change. While adsorption takes about 30 min, the conformational changes happens over 180 min.

One of the theories discussed in the literature deals explicitly with charges on the surface of the nanoparticle, as well as the charge of the protein. We therefore had to check if the interaction of protein and nanoparticle is driven purely by electrostatic interaction, or by other factors as well. To test this we made adsorption experiments in the presence of salt, which should interrupt electrostatic interaction between protein and particle and result in lower binding. The addition of 1 M salt into the buffer prior to the adsorption experiment resulted for myoglobin in a decrease of binding capacity down to 46 ± 8%. This shows that electrostatic interaction is not the only force, but contributes a very significant portion to the overall force between myoglobin and the nanoparticle. BSA shows hydrophobic interaction with the nanoparticle as the adsorption capacity increased to 126 ± 22% when adding salt to the mixture. Interestingly, both proteins show different interaction mechanism (mixed mode for myoglobin and hydrophobic for BSA) but show the same pattern regarding the size dependency of the conformational change.

We also performed desorption experiments of adsorbed protein, but were not able to desorb any protein after the adsorption and conformational change of the protein, even under high salt conditions. Unfortunately our method did not allow to desorb the proteins before they changed conformation due to the handling time of samples. We can conclude from these experiments that there is an additional hydrophobic contribution of binding energy once the protein changes its conformation on the surface. Whether this additional hydrophobic binding energy is the driving force of the conformational change or rather a consequence of the unfolding is impossible to tell with the currently available data.

### 3.5 Evaluation of common theories

A number of theories have been proposed to explain the particle size‐dependent conformational change of proteins after adsorption. The most accepted explanation is that the curvature of the particle itself leads to a change in either interaction area of protein and particle, or to a bending of the protein to accommodate the curvature of the particle. This explanation is usually used for very small particles and may be a factor on very small scales, but has to be reevaluated to see if it can explain the conformational changes seen in this study.

Another explanation was based on the electrostatic interaction between protein and nanoparticle. The double‐layer potential build up at the interface of particle and solution is geometry‐dependent and therefore dependent on the particle size. We calculated the implications of these models and tried to find something that was able to explain the pattern we saw in our experiments: no change between 30 and 100 nm particle size, significant change between 100 and 300 nm size, and no change between 300 and 1000 nm particle size.

### 3.6 Calculation of interaction area based on nanoparticle size

We defined the interaction area of nanoparticle and protein to be the portion of the protein surface that is closer than 0.4 nm to the surface of the particle, which is a typical interaction range of electrostatic interaction 26. For the case of hydrophobic interaction there is no interaction area to be calculated with this model, as hydrophobic interaction acts only on contact. Equations for calculation and figures for explanations can be seen in the supporting information. Longer interaction ranges than 0.4 nm would be a case of electrostatic interaction in a solution of lower conductivity. Shorter interaction would represent Van der Waals interactions, hydrophobic interactions have no range by themselves, but typically act on a range of 0.1 nm or lower through the exclusion of water.

**Figure 5:**
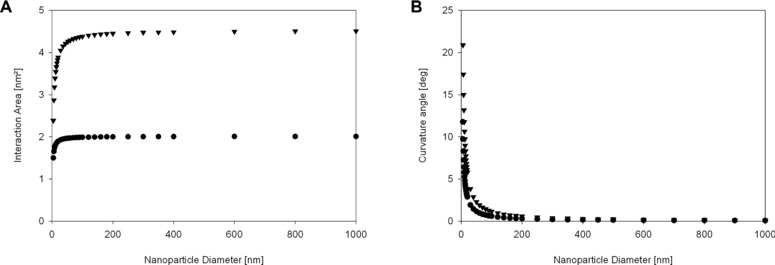
(A) Interaction area calculated for two differently sized theoretical proteins, 2 (circle) and 4 nm (triangle) and (B) the angle of this interaction. No significant change in interaction area or angle of interaction is visible for particles bigger than 150 nm.

The interaction area on the protein given in square nanometer dependent on the nanoparticle size is presented in Fig. 5A for the case of 0.4 nm interaction range (equations and additional interaction ranges can be found in the Supporting information, Fig. S5). From this graph, we can see that the interaction area is steeply increasing for smaller particle sizes and no significant change is observed for particles larger than 100 nm for both a model protein of 2 nm size and a model protein of 4 nm size. We conclude that the size dependency of the interaction area might have a large impact on very small particles below 50 nm but the effect is negligible for particles above 100 nm.

### 3.7 Calculation of an angle of interaction based on nanoparticle size

Another hypothesized explanation for the direct influence of the curvature on the conformation of the adsorbed protein is that the protein has to bend to accommodate the curved surface. This explanation is typically used in studies presenting an inverse dependency of size and conformational change of the adsorbed proteins. We calculated the angle between the point at which the two spheres touch and the point on the particle circumference corresponding to the projected area (the shadow) of the protein. Equations and figures for explanation can be seen in the Supporting information, Fig. S6. Figure 5B shows the calculated angle in relation to the particle size for two sizes of protein (2 and 4 nm). The angle is steeply decreasing for very small particles and might be an explanation for changes seen in this particle range, but does not significantly change for particles above 100 nm.

### 3.8 Calculation of double‐layer potential based on nanoparticle size

**Figure 6:**
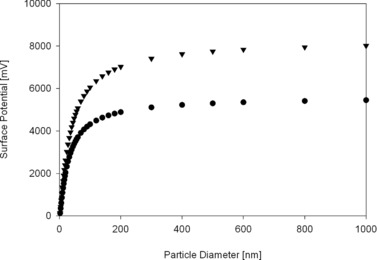
Surface potential calculated for hypothetical proteins of 2 (circle) and 4 nm (triangle) in diameter.

An indirect influence of the surface curvature, hypothesized as reason for size‐dependent denaturation by Vertegel et al. 26, is the geometry‐dependent double‐layer potential. This potential builds up at the interface between the solid particle and the liquid buffer and is dependent on the size of the particle, increasing with the particle diameter. Larger particles could lead to a stronger electrostatic force and this could result in conformational changes in the protein. Figure 6 shows the potential in millivolt calculated by the equations presented by Vertegel et al. As in the other proposed theories, we see a steep increase for small particles, but a shallower curve. The change in double‐layer potential is quite significant for smaller particles than 100 nm and might therefore not be distinguishable from other factors such as interaction area and curvature. This change in double‐layer potential is negligible above a particle size of 300 nm. This leaves a window of 100–300 nm particle size where the direct contributions of the surface (curvature and interaction) are negligible, and the potential is still changing. We expect that any change seen in this size range might be at least partially attributed to the change in the double‐layer potential.

## 4 Concluding remarks

We studied the interaction of nanoparticles with nine proteins and demonstrated conformational changes for myoglobin and BSA upon adsorption to nanoparticles. The susceptibility of proteins to conformational changes upon adsorption varies substantially for different protein‐nanoparticle combinations and might be specific for any combination. All proteins that showed conformational change (myoglobin and BSA) did this in a nanoparticle size‐dependent manner. Both proteins showed conformational changes on nanoparticle sizes bigger than 150 nm, but no significant change in conformation for any smaller size. Although the interaction mechanism and the kinetics of the conformational change is different for both proteins (mixed mode for myoglobin and hydrophobic for BSA), they show the same pattern of conformational change. Using model calculations we excluded surface curvature as the major driving force for the size ranges in which conformational changes occurred in this study (above 100 nm). Geometry‐dependent double‐layer potentials may still play a significant role for the denaturation of myoglobin, as it interacts in a mixed mode. For the binding of BSA, the double‐layer potential should be insignificant as it is purely hydrophobic interactions. This interesting result still asks the question if some of the behaviors are universal, or may be specific for these two proteins. It also asks the question if we are missing a mechanism completely that might explain the behavior of both BSA and myoglobin. For both proteins, the conformational change was particle size‐dependent. It would be interesting if this is universally true for all proteins susceptible to denaturation on these particles, or if proteins exist which show denaturation, but not in a particle size‐dependent way. If all proteins that do show conformational change show the same pattern, regardless of the specific protein or of the interaction force between them, we might be able to construct a new theory to explain this phenomenon.

**Practical application**

Nanomaterials are under heavy investigation for medical and engineering applications, but little is known about the interaction between nanoparticles and proteins. Here, we investigated nanoparticle size‐dependent denaturation of adsorbed proteins and the use of CD to study such changes. Using model calculations, we showed the lacking explanatory power of commonly used theories. Furthermore, we provided the first solid dataset for a variety of proteins and a whole range of nanoparticle sizes. This enables the design of nanoparticles that show low size‐dependent denaturation of the adsorbed protein for various applications ranging from downstream applications and nanosensors to medical usage. In all applications where nanoparticles are used to bind protein, one has to be aware of this effect if the structure of the protein has to be preserved.

*The authors have declared no conflict of interest*.

## Supporting information

As a service to our authors and readers, this journal provides supporting information supplied by the authors. Such materials are peer reviewed and may be re‐organized for online delivery, but are not copy‐edited or typeset. Technical support issues arising from supporting information (other than missing files) should be addressed to the authors.

Supporting Material
