# Supplementary material for: Protein adsorption onto nanoparticles induces conformational changes: Particle size dependency, kinetics, and mechanisms
Source: Eng Life Sci. 2015 Nov 10;16(3):238–46. doi: 10.1002/elsc.201500059 (PMC4949706; doi:10.1002/elsc.201500059)
Supplement: Supplementary file 1 — Supporting Material [file ELSC-16-238-s001.pdf]

Supporting Information

Protein adsorption onto nanoparticles induces conformational changes: Particle size dependency, kinetics and mechanisms

Peter Satzer,[a] Frantisek Svec,[b] Gerhard Sekot,[c] and Alois Jungbauer [a,c] *

[a] Department of Biotechnology, University of Natural Resources and Life Sciences Vienna (BOKU), Muthgasse 18, 1190 Vienna, Austria, [b] Lawrence Berkeley National Laboratory, The Molecular Foundry, Berkeley, CA 94720, USA, [c] Austrian Centre of Industrial Biotechnology (ACIB), Muthgasse 18, 1190 Vienna, Austria

* Address correspondence to

alois.jungbauer@boku.ac.at
Muthgasse 18, 1190 Vienna
Telephone: +43476546226
Fax: +43476546677

Relevant publications dealing with denaturation of proteins upon adsorption to nanoparticles – Supporting Information Table S1

List of some relevant publications for the topic of particle size dependent denaturation of proteins upon adsorption and if they indicated size dependency and/or conformational change.

Type	*Dimension*	*Protein*	*Material*	*Conformational Change?*	*Size dependency*	*Group*	*Methods*	*Reference*
Particle	9 nm	Lysozyme	Silica	Yes	ND	1998, Tian	Circular dichroism	[1]
Particle	5-100 nm	Histone, albumin, insulin, globulin, fibrinogen	Gold	Yes	Yes	2009, Lacerda	Circular dichroism, Fluorescence	[2]
Particle	25 nm	Apolipoprotein, HDL, HAS	Polystyrene	Yes	ND	2011, Cukalevski	Circular dichroism, Fluorescence, Limited proteolysis	[3]
Crystal	unknown	Myoglobine	Hydrotalcite (NiAl)	Yes	Yes	2009, Bellezza	FT-IR, Fluorescence, Raman, Activity	[4]
Particle	100-200 nm	Lysozyme	Polystyrene +polylactic acid	ND	ND	2008, Cai	TEM, SEM, AFM, DSC	[5]
Particle	20, 30 nm	butyrylcholinesterase	Silica	Yes	Yes	2010, Wang	Activity	[6]
Particle	10-60 nm	butyrylcholinesterase	Metal	Yes	Yes	2010, Wang	Activity	[6]
Particle	60-600 nm	Blood plasma proteins	Polystyrene	ND	Yes	1998, Lück	2D-DIGE	[7]
Particle	90 nm	Beta-lactoglobulin	Silica	Yes	ND	2008, Wu	Fluorescence, FTIR, CD	[8]
Surface	-	Human carbonic anhydrase II	Functionalized thiols	Yes	ND	2005, Karlsson	Surface plasmon resonance	[9]
Particle	35, 120, 140 nm	Blood plasma proteins	Silica	ND	Yes	2013, Tenzer	SDS-PAGE,	[10]
Particle	4, 15 nm	Ribonuclease A	Silica	Yes	Yes	2007, Shang	Circular Dichroism	[11]
Particle	4, 20, 100 nm	Lysozyme	Silica	Yes	Yes	2004, Vertegel	Circular Dichroism, Activity	[12]
Particle	4, 15 nm	Acylphosphatase	Silica	Yes	Yes	2013, Shrivastava	Activity, Circular dichroism, NMR	[13]
Particle	6, 9, 15 nm	Human carbonic anhydrase I and II	Silica	Yes	Yes	2004, Lundqvist	Circular dichroism, NMR	[14]
Particle	9 nm	Human carbonic anhydrase II	Silica	Yes	ND	1997, Billsten	Circular dichroism, Fluorescence	[15]
Particle	9 nm	Human carbonic anhydrase I	Silica	Yes	ND	2005, Lundqvist	NMR	[16]
Particle	6, 9 nm	Human carbonic anhydrase I and II	Silica	Yes	No	2005, Lundqvist	NMR	[17]
Particle	6 nm	Chymotrypsin	Functionalized gold	Yes	ND	2002, Fischer	Circular dichroism, Activity	[18]
Particle	9 nm	Lysozyme	Silica	Yes	ND	1999, Bower	Circular dichroism, Activity	[19]
Particle	4.5 nm	Human carbonic anhydrase XII	Quantum dot	Yes	ND	2010, Manokaran	Fluorescence, Activity	[20]
Particle	<50nm	Bovine serum albumin	Al2O3	Yes	ND	2014, Rajeshwari	FT-IR, Circular dichroism, Fluorescence, UV-VIS	[21]
Particle	15, 260nm	TNF-α, IL-8	Carbon black	ND	Yes	2010, Brown	Cellular uptake	[22]
Particle	10 kDa	Lysozyme, IgG	Fullerol	Yes	ND	2014, Chen	Circular Dichroism, Fluorescence, DSC	[23]
Particle	8, 45 nm	Plants	Silver	ND	Yes	2014, Syu	Plant growth	[24]
Particle	30. 200. 400 nm	Blood plasma proteins	Fe3O4	ND	Yes	2014, Hu	2D DIGE	[25]
Particle	5-50	Ovalbumin	Silver	Yes	ND	2014, Joshi	UV-VIS, Raman Spectroscopy	[26]
Particle	20, 40, 80 nm	Tetanus Oxois	Gold	Yes	Yes	2014, Barhate	Circular Dichroism, Thermodynamic studies	[27]

Protein Characteristics - Supporting Information Table S2 and Table S3

In our study we investigated 9 model proteins, which are presented in supporting information Table S2. We used a variety of different model proteins to cover a wide range of different protein characteristics, namely size ranging from 11 kDa to 160 kDa, a pI ranging from 4.2 to 11.0 and different stabilities represented by different melting temperatures ranging from 44 °C to 85 °C. Two of this model proteins did not show significant binding: chymotrypsin and glucose oxidase and so they were excluded from further studies. All other proteins were investigated for their structural composition (supporting information Table S3).

Table S2 – List of Proteins Used in this Study and their Characteristics

Protein	MW, kDa [a]	pI [b]	T_m, °C [c]	binds to 70 nm nanoparticles
lysozyme	11	11.0	73	Yes
cytochrome c	12	10.3	85	Yes
ribonuclease A	13	9.6	64	Yes
myoglobin	17	6.8	80	Yes
β casein	25	4.7	70	Yes
ovalbumin	42	4.7	84	Yes
bovine serum albumin	66	4.7	62	Yes
chymotrypsin	25	8.7	44	No
glucose oxidase	160	4.2	56	No

[a] Molecular weight of the protein, [b] Isoelectric point, [c] Melting point representing thermal stability.

Table S3 – Structural Composition of Proteins Determined by Circular Dichroism and concentration independent Structure Prediction

Protein	content of structures, %		
	α-helical	β-sheet	random-coil
lysozyme	32.6 +/- 0.5 %	13.8 +/- 0.5 %	34.1 +/- 0.1 %
cytochrome c	28.2 +/- 0.4 %	15.5 +/- 0.6 %	33.2 +/- 0.1 %
ribonuclease a	17.6 +/- 0.3 %	25.9 +/- 0.5 %	36.2 +/- 0.1 %
myoglobin	55.2 +/- 0.9 %	4.2 +/- 0.2 %	26.9 +/- 0.1 %
β casein	7.1 +/- 0.4 %	31.8 +/- 0.4 %	41.2 +/- 0.1 %
ovalbumin	42.9 +/- 1.0 %	13.9 +/- 0.4 %	29.3 +/- 0.2 %
bovine serum albumin	42.3 +/- 0.3 %	12.01 +/- 0.2 %	30.6 +/- 0.2 %

CD Measurement Raw Data - Supporting Information Figure S1

Using our method of compiling 10 individual CD spectra to one spectra before using it for structure determinations we were able to obtain high quality CD spectra despite the unavoidable fact of signal loss due to the particles in solution. Figure S1 shows such a spectra from BSA bound 200 nm particles. We can see that using 10 individual measurements condensed to one for further investigation results in good quality spectra without additional smoothing of the spectra.

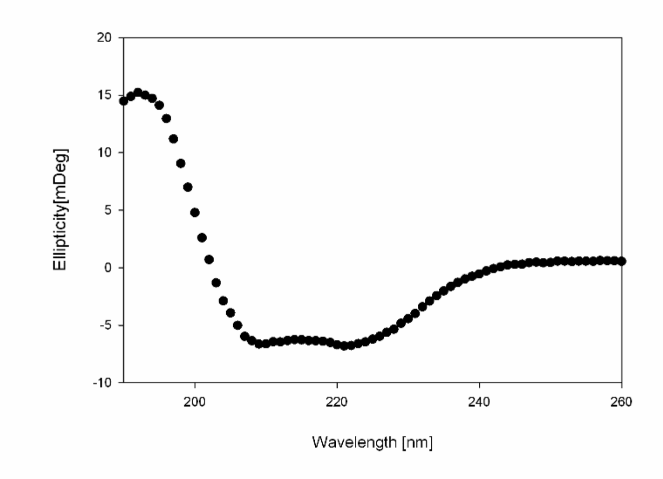

Figure S1 CD-spectra of BSA adsorbed onto 200 nm silica nanoparticles.

Protein Adsorption to Nanoparticles - Supporting Information Figure S2 and Figure S3

From the model proteins we investigated, 3 did not show any significant change in secondary structure upon adsorption to nanoparticles (supporting information Figure S2), namely beta casein, lysozyme and ribonuclease A. These 3 proteins were not further studied. For 2 model proteins we saw a trend in the data (supporting information Figure S3) but the standard deviations from the measurements are too big to draw a final conclusion about whether there is a change in structure or not.

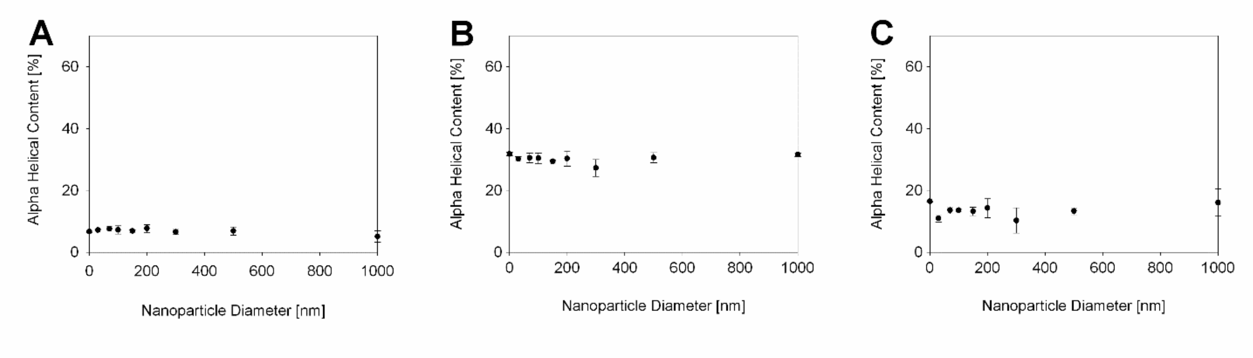

Figure S2 α-Helical content of β casein (A), lysozyme (B) and ribonuclease A (C) adsorbed on nanoparticles varying in sizes. The point at 0 nm nanoparticle diameter is the structure of the protein in solution with no nanoparticles. 10 individual spectra were condensed into one before structure determination. 3 of such sets were used to calculate standard deviations shown in the error bars of the figure.

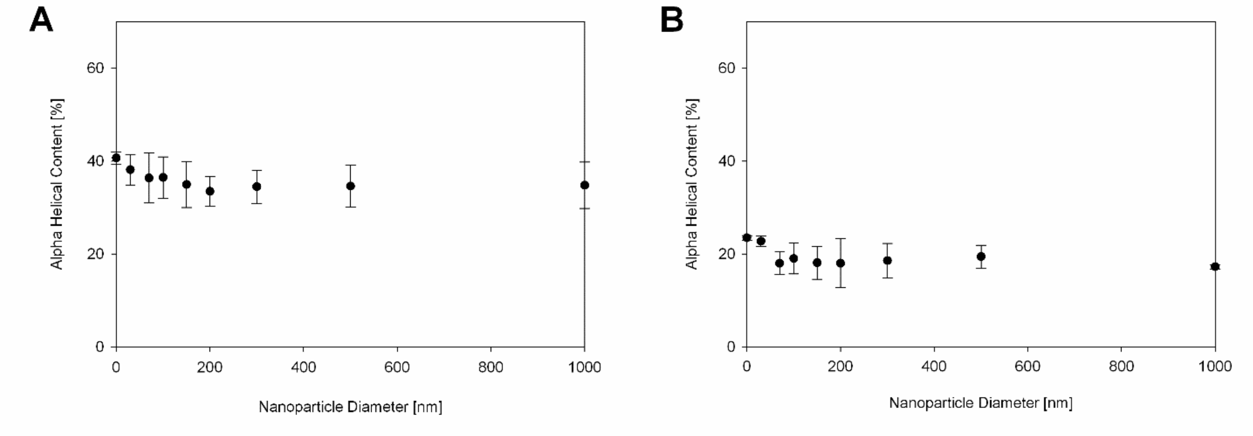

Figure S3. α-Helical content of ovalbumin (A) and cytochrome C (B) adsorbed on nanoparticles varying in sizes. The point at 0 nm nanoparticle diameter is the structure of the protein in solution with no nanoparticles. 10 individual spectra were condensed into one before structure determination. 3 of such sets were used to calculate standard deviations shown in the error bars of the figure.

Adsorption Isotherms of all Particle Sizes for Myoglobine and BSA. Supporting Information Figure S4

We determined the adsorption isotherms for all particles by simple adsorption experiments done in triplicates, curves of 30, 100, 300 and 1000 nm are shown (Figure S4). This curves were only used for determining the protein amount on the particle.

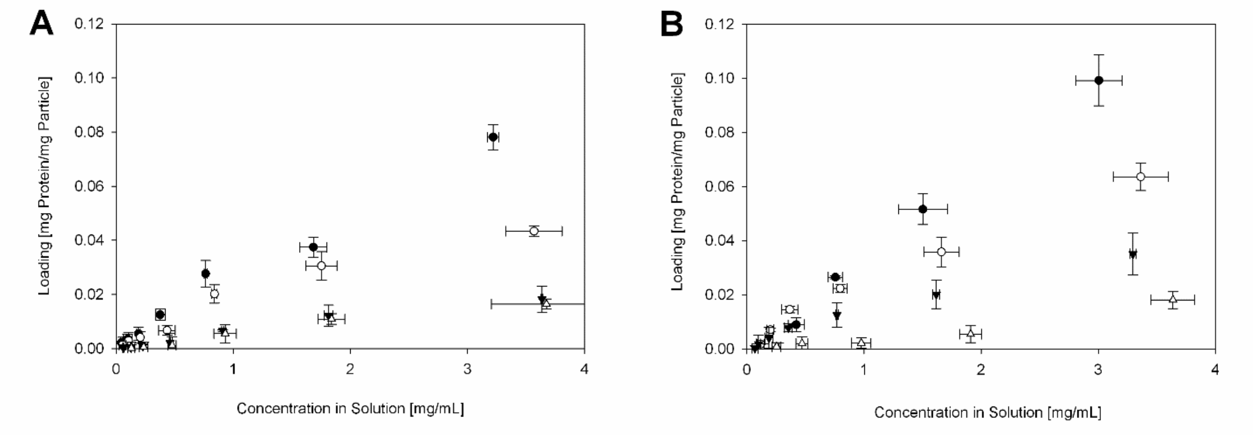

Figure S4 Adsorption isotherms of myoglobin (A) and bovine serum albumin (B) on 30 (●), 100 (○), 300 (▼)) and 1000 nm (Δ) silica nanoparticles. We present mean values and standard deviations from three independent measurements.

Determination of available surface for interaction between protein and nanoparticle – Supporting information Figure S5

For evaluation of the curvature based theory we calculated the cross-section available for interaction between protein and nanoparticle using an interaction cutoff of 0.4 nm, a usual penetration length of electrostatic forces in buffered solutions. We calculated this cross-section for differently sized nanoparticles and two different sizes of proteins, 2 nm and 4 nm sized spherical proteins. Equations (1) to (3) allow the calculation of the radius of the interaction area $\boldsymbol{r}$ which directly gives the interaction area using $\boldsymbol{A = \pi r^2}$.

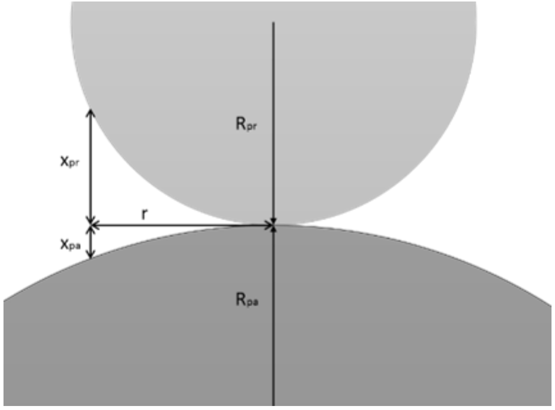

Figure S5a – Graphical representation of two touching spheres and the important system defining lengths used in Equation (1) to (3) to calculate the radius of the interaction area.

$$x_{pa} + x_{pr} = 0.4 \quad (1)$$

$$R_{pa}^2 = r^2 + (R_{pa} - x_{pa})^2 \quad (2)$$

$$R_{pr}^2 = r^2 + (R_{pr} - x_{pr})^2 \quad (3)$$

The calculations were done with an interaction range of 0.1 nm, 0.4 nm (as shown in the equations) and 0.8 nm (Figure S5b Panel A-C). The different interaction ranges do not show any significant impact on the shape of the curve.

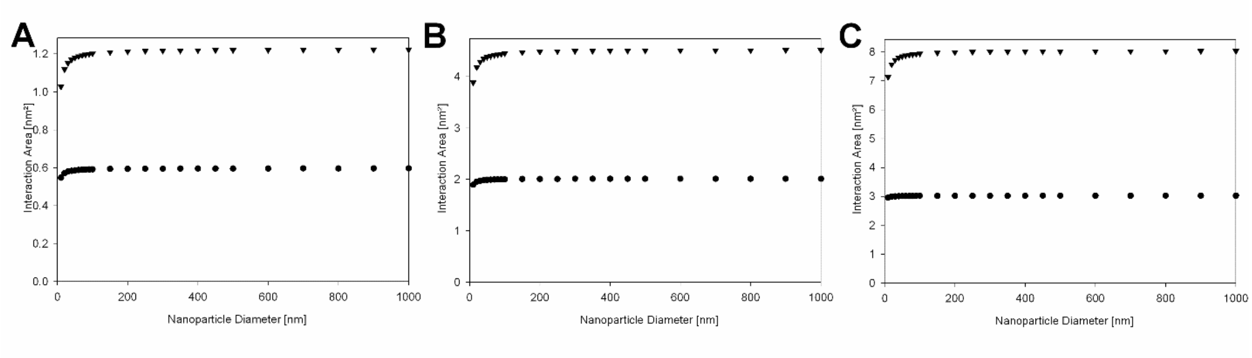

Figure S5b – Interaction area of two touching spheres with an interaction range of 0.1 nm (Panel A), 0.4 nm (Panel B) and 0.8 nm (Panel C) calculated by the equations (1) to (4).

Determination of surface curvature as seen by a protein sitting on top of a nanoparticle – Supporting information Figure S6

A second method for determining the curvature seen by the protein adsorbed to the nanoparticle is to calculate the angle at which the protein would have to bend to accommodate the surface presented to the protein. We calculated this angle for small and large proteins (2 nm and 4 nm) size as depicted in Figure S2 and the corresponding Equations (4) to (5).

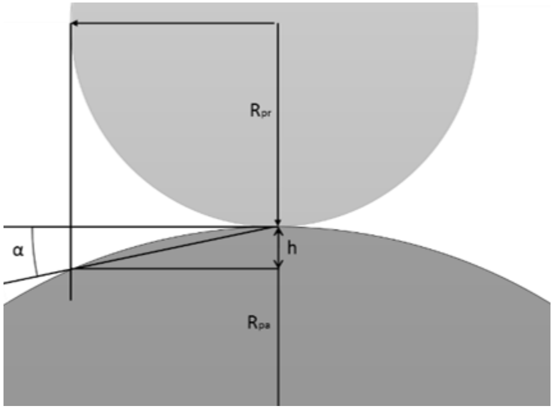

Figure S6 – Graphical representation of two touching spheres and the important system defining lengths used in Equation (4) to (5) to calculate the angle at which the protein would have to bend to accommodate the surface.

$$R_{pa} = \frac{4h^2 + (2R_{pr})^2}{8h} \qquad (4)$$

$$\tan\alpha = \frac{h}{R_{pr}} \qquad (5)$$

References

1. Tian, M., et al., *Structural Stability Effects on Adsorption of Bacteriophage T4 Lysozyme to Colloidal Silica.* Journal of Colloid and Interface Science, 1998. **200**(1): p. 146-154.
2. Lacerda, S.H.D.P., et al., *Interaction of Gold Nanoparticles with Common Human Blood Proteins.* ACS Nano, 2009. **4**(1): p. 365-379.
3. Cukalevski, R., et al., *Structural Changes in Apolipoproteins Bound to Nanoparticles.* Langmuir, 2011. **27**(23): p. 14360-14369.
4. Bellezza, F., et al., *Structure and Catalytic Behavior of Myoglobin Adsorbed onto Nanosized Hydrotalcites.* Langmuir, 2009. **25**(18): p. 10918-10924.
5. Cai, C., et al., *Charged Nanoparticles as Protein Delivery Systems: A Feasibility Study Using Lysozyme as Model Protein.* European Journal of Pharmaceutics and Biopharmaceutics, 2008. **69**(1): p. 31-42.
6. Wang, Z., et al., *Adsorption and inhibition of butyrylcholinesterase by different engineered nanoparticles.* Chemosphere, 2010. **79**(1): p. 86-92.
7. Lück, M., et al., *Analysis of Plasma Protein Adsorption on Polymeric Nanoparticles with Different Surface Characteristics.* Journal of Biomedical Materials Research, 1998. **39**(3): p. 478-485.
8. Wu, X. and G. Narsimhan, *Characterization of Secondary and Tertiary Conformational Changes of β-Lactoglobulin Adsorbed on Silica Nanoparticle Surfaces.* Langmuir, 2008. **24**(9): p. 4989-4998.
9. Karlsson, M., et al., *Reduction of Irreversible Protein Adsorption on Solid Surfaces by Protein Engineering for Increased Stability.* Journal of Biological Chemistry, 2005. **280**(27): p. 25558-25564.
10. Tenzer, S., et al., *Rapid formation of plasma protein corona critically affects nanoparticle pathophysiology.* Nat Nano, 2013. **8**(10): p. 772-781.
11. Shang, W., et al., *Unfolding of Ribonuclease A on Silica Nanoparticle Surfaces.* Nano Letters, 2007. **7**(7): p. 1991-1995.
12. Vertegel, A.A., R.W. Siegel, and J.S. Dordick, *Silica Nanoparticle Size Influences the Structure and Enzymatic Activity of Adsorbed Lysozyme.* Langmuir, 2004. **20**(16): p. 6800-6807.
13. Shrivastava, S., et al., *Identifying Specific Protein Residues That Guide Surface Interactions and Orientation on Silica Nanoparticles.* Langmuir, 2013. **29**(34): p. 10841-10849.
14. Lundqvist, M., I. Sethson, and B.-H. Jonsson, *Protein Adsorption onto Silica Nanoparticles: Conformational Changes Depend on the Particles' Curvature and the Protein Stability.* Langmuir, 2004. **20**(24): p. 10639-10647.
15. Billsten, P., et al., *Adsorption to silica nanoparticles of human carbonic anhydrase II and truncated forms induce a molten-globule-like structure.* FEBS Letters, 1997. **402**(1): p. 67-72.
16. Lundqvist, M., I. Sethson, and B.-H. Jonsson, *Transient Interaction with Nanoparticles "Freezes" a Protein in an Ensemble of Metastable Near-Native Conformations†.* Biochemistry, 2005. **44**(30): p. 10093-10099.
17. Lundqvist, M., I. Sethson, and B.-H. Jonsson, *High-Resolution 2D 1H−15N NMR Characterization of Persistent Structural Alterations of Proteins Induced by Interactions with Silica Nanoparticles.* Langmuir, 2005. **21**(13): p. 5974-5979.
18. Fischer, N.O., et al., *Inhibition of Chymotrypsin through Surface Binding Using Nanoparticle-based Receptors.* Proceedings of the National Academy of Sciences, 2002. **99**(8): p. 5018-5023.
19. Bower, C.K., et al., *Activity losses among T4 lysozyme charge variants after adsorption to colloidal silica.* Biotechnology and Bioengineering, 1999. **64**(3): p. 373-376.

20. Manokaran, S., et al., *Differential modulation of the active site environment of human carbonic anhydrase XII by cationic quantum dots and polylysine.* Biochimica et Biophysica Acta (BBA) - Proteins and Proteomics, 2010. **1804**(6): p. 1376-1384.
21. Rajeshwari, A., et al., *Spectroscopic studies on the interaction of bovine serum albumin with Al2O3 nanoparticles.* Journal of Luminescence, 2014. **145**(0): p. 859-865.
22. Brown, D.M., et al., *Interaction between nanoparticles and cytokine proteins: impact on protein and particle functionality.* Nanotechnology, 2010. **21**(21): p. 215104-215112.
23. Chen, P., et al., *Contrasting effects of nanoparticle binding on protein denaturation.* Journal of Physical Chemistry C, 2014. **118**(38): p. 22069-22078.
24. Syu, Y.Y., et al., *Impacts of size and shape of silver nanoparticles on Arabidopsis plant growth and gene expression.* Plant Physiology and Biochemistry, 2014. **83**: p. 57-64.
25. Hu, Z., et al., *Nanoparticle size matters in the formation of plasma protein coronas on Fe3O4 nanoparticles.* Colloids and Surfaces B: Biointerfaces, 2014. **121**: p. 354-361.
26. Joshi, D. and R.K. Soni, *Laser-induced synthesis of silver nanoparticles and their conjugation with protein.* Applied Physics A: Materials Science and Processing, 2014. **116**(2): p. 635-641.
27. Barhate, G.A., et al., *Structure function attributes of gold nanoparticle vaccine association: Effect of particle size and association temperature.* International Journal of Pharmaceutics, 2014. **471**(1-2): p. 439-448.
